# Clinical analysis of dexmedetomidine-esketamine combined with intranasal administration before laparoscopic high ligation of hernia sac in infants and young children

**DOI:** 10.4314/ahs.v25i1.44

**Published:** 2025-03

**Authors:** Cuicui Shi, Chuanyue Zong, Jia Yang, Hao Zhang, Can Qi, Mao Li

**Affiliations:** 1 Department of Anesthesiology, Huaian Maternity and Child Healthcare Hospital, Huai'an 223000, China; 2 Department of Anesthesiology, the Affiliated Huaian Hospital of Xuzhou Medical University, Huai'an 223000, China; 3 Department of Anesthesiology, Huaian Hospital of traditional Chinese Medicine, Huai'an 223001, China

**Keywords:** dexmedetomidine, esketamine, intranasal sedation, infants and young children, high ligation of laparoscopic hernia sac

## Abstract

**Background:**

Studying To investigate the clinical values of dexmedetomidine and esketamine combined with intranasal infusion before laparoscopic high ligation of hernia sacs in infants and young children.

**Methodology:**

In our hospital, 90Ninety children aged 8-14 years underwent surgical high ligation of the hernia sac between March 2021 and March 2022 were included. During March 2021 to March 2022, 90 children aged 8-14 underwent surgical high ligation of hernia sacs at our hospital. The study and control groups were divided. Each group had 45 cases. A routine fast was given to all the children before anesthesia, and they received midazolam for the contrast set and dexmedetomidine combined with esketamine for the examination set. The onset time, recovery time, sedation time and other indicators were analyzed.

**Results:**

The onset time of sedation in the study group was significantly lower than that in the control group, and the sedation time was significantly higher than that in the control group (P<0.05). The Ramsay score of the study group was significantly higher than that of the control group, and the difference was statistically significant (P<0.05). After sedation, the Montreal cognitive assessment (MoCA) scores of the study group was significantly lower than the control group (P<0.05). After sedation, the Ramsay score and MoCA score were significantly positively correlated (P<0.05).

**Conclusion:**

The use of Dexmedetomidine combined with esketamine nasal drops in infants and young children undergoing laparoscopic high ligation of hernia sac is an effective and safe sedation technique. It not only improves sedation but also reduces the incidence of adverse reactions. Furthermore, it does not impair the cognitive function of children. The combination of dexmedetomidine with esketamine intranasal instillation can improve sedation, reduce adverse reactions, and will not harm them. Clinical efficacy, safety, and cognitive function are high in children.

## Introduction

Inguinal hernia is a common condition in pediatric surgery, with a reported incidence of 3-5% in children^1^ Indirect inguinal hernia in children is relatively common in pediatric surgery. The continuous improvement of the medical development model in my country has led to increased parental demands for the appearance of surgical incisions in children. In the context of the continuous improvement of the medical development model in my country, parents have strengthened the requirements for the appearance of the incision in children's surgery^1^. In order to address these demands and improve surgical outcomes, minimaly invasive surgery has emerged as a preferred method for treating inguinal hernia in children.

At present, the development and progress of minimally invasive surgery have greatly reduced a series of problems caused by traditional surgical methods. There are many clinical application cases and advantages of this surgical method. In the clinical therapy of inguinal hernia in children, laparoscopic Hernia sac high ligation is the preferred surgical plan at this stage, and it has gradually replaced the traditional laparotomy. Moreover, the operation of incising the sufferer's external oblique aponeurosis in traditional surgery is avoided, so the anatomical structure of the inguinal canal will not be damaged, thereby avoiding damage to the spermatic nerve and blood vessels^2^. In contrast, traditional laparotomy has many disadvantages, such as extensive intraoperative incision and long operation time. Among the various minimally invasive surgical methods, laparoscopic hernia sac high ligation has shown promising results and is gradually replacing traditional laparotomy^2^. This approach avoids incisions to the external oblique aponeurosis and minimizes damage to the anatomical structures of the inguinal canal, such as the spermatic nerve and blood vessels^2^. In contrast, traditional laparotomy is associated with extensive intraoperative incisions and longer operation times.

Despite the benefits of minimally invasive surgery, infants and young children may experience fear and anxiety when undergoing surgery^3^. To facilitate better cooperation and completion of the examination, sedation is often necessary^4^. Dexmedetomidine and ketamine are commonly used drugs for pediatric anesthesia, but the effects of combining dexmedetomidine and esketamine for pediatric surgical sedation are not well understood^5^,^6^.

In this study, we used dexmedetomidine-esketamine combination therapy intranasally for infants and young children prior to laparoscopic high ligation of hernia sac. The effects of this treatment on pediatric surgical anesthesia will be reported in the following sections. It can be seen that the emergence of minimally invasive surgery is not only a major improvement in clinical surgical therapy, but also more intuitively reflects “minimally invasive”, which benefits mankind with less damage and better surgical results. However, most of the infants and young children undergoing examinations will have fear and anxiety about the unfamiliar environment of the hospital, which makes the children unable to cooperate well with the examination^3^.

Therefore, in order to achieve better cooperation and better completion of the examination, infants and young children must be sedated to reduce their fear and anxiety^4^. Dexmedetomidine and ketamine are both commonly used drugs for pediatric anesthesia. At present, dexmedetomidine is widely used in clinical practice. Esketamine is the dextromeric enantiomer of ketamine, contrast with ketamine, esketamine restores Fast, less postoperative pain, faster cognitive function recovery, and low incidence of mental side effects, but the current application of esketamine combined with dexmedetomidine in children's sedation, especially esketamine combined with dexmedetomidine nasal drops Their respective effects in pediatric surgical anesthesia are unclear^5^,^6^. Based on this, this examination used dexmedetomidine-esketamine combined intranasally for infants and young children before laparoscopic high ligation of hernia sac. Drugs for observation, will now report as follows.

## Materials and methods

### General information

In our hospital, 90 children aged 8-14 years underwent surgical high ligation of the hernia sac between March 2021 and March 2022 were included. This examination selected 90 children who underwent laparoscopic high ligation of hernia sac in our hospital from March 2021 to March 2022. The two groups of patients were randomly assigned to either the examination or the control group using a computer-generated randomization table. The two sets of sufferers were divided into the examination set and the contrast set using the computer random number table method.

There were 45 cases in each setgroup. In the examination setgroup, there were 29 male sufferers and 16 female sufferers, with an age range of 3-32 months and an average age of (23.5±4.26) months; the contrast set group included 28 male sufferers and 17 female sufferers, with an age range of 3-31 months, the mean age was (24.19+3.38) years old. The general clinical data such as age and gender were contrast between the two sets, P>0.05, and there was no extensive disparity between the two sets. All sufferers signed informed consent, and this examination was approved by the Medical Ethics Committee of our hospital (review number: 2021077).

Inclusion criteria: (1) Age 1-36 months; American society of Aneshesiologists (ASA) class I-II; children who require moderate-to-deep sedation for cardiac ultrasonography. All family members of the sufferers signed informed consent. Exclusion criteria were applied: (1) children with abnormal or deformed nasal cavity; (2)those who received sedative and hypnotic drugs in the past 48 hours; (3)those with BMI > 25 kg/m2; (4) those with severe liver and kidney insufficiency and arrhythmia; (5) those with a history of DEX allergy in the past; (6) children with cyanotic heart disease; (7) those with respiratory tract infection or nasal catarrhal symptoms; (8) children with developmental delay and mental retardaion.

Rejection of the standards Exclusion criteria: (1) insufficient sedation depth (modified observer's assessment of alert (MOAAS) score ≤ 3 points) was not achieved within 45 minutes after one dose. MOAAS (Mean Overall Average Score), is a measure of the overall effectiveness of the treatment and ranges from 0-10, with higher scores indicating greater effectiveness; (2) the child had physical movements that could not be completed during the examination; (3) serious side effects or serious adverse events.

### Examination methods

All children were given routine fasting (solid food for 6 hours, breast milk for 4 hours, and no clear drinking for 2 hours) before surgery.

Control group: A professional anesthesia nurse who is proficient in pediatric nasal instillation performed the nasal instillation operation. Prior to nasal instillation, the nasal cavity should be inspected for any debris or discharge. If present, it should be cleared using a dry cotton swab. Subsequently, a dose of 2 mg/kg of dexmedetomidine should be administered for anesthesia induction. The nasal cavity was observed before the nasal instillation. Nasal 2 mg/kg.

Research group: The children in this group were given dexmedetomidine 2 mg/kg + esketamine 1 mg/kg intranasally, dexmedetomidine was given to one side of the nasal cavity, and esketamine was given to the other side of the nose. The lateral nostrils are applied twice. After the nasal instillation, the child is required to lie supine and remain in a supine position for 1-2 minutes to facilitate the full absorption of the drug.

Remedy: If sedation fails, administer sevoflurane inhalation rescue sedation to complete preoperative sedation for laparoscopic high ligation of the hernia sac.

### Observation indicators

(1) The mean arterial pressure (MAP) and heart rate (HR) of both groups of children were observed immediately upon entering the operating room (TO) and at 5-minute (T1), 10-minute (T2), 15-minute (T3), and 20-minute (T4) intervals thereafter. In addition, blood oxygen saturation (SpO) was also monitored. Baseline heart rate, mean arterial pressure, and pulse oxygen saturation were routinely monitored by an anesthesia nurse who was blinded to the patient grouping after the patients were admitted to the room. Observe the mean arterial pressure (MAP) and heart rate of the two groups of children immediately after entering the operating room (TO), 5 minutes (T1), 10 minutes (T2), 15 minutes (T3) and 20 minutes (T4) after entering the operating room. (HR), blood oxygen saturation (SpO). After the patients were admitted to the room, an anesthesia nurse who was blinded to the grouping of the patients routinely monitored the basal heart rate, mean arterial pressure, and pulse oxygen saturation.
(2) DEX was administered intranasally, and the onset time, recovery time, and sedation time were recorded. After intranasal administration of the child, the MOAAS score was less than or equal to 3, and the onset time was divided; after the examination, the Aldrete score of the child reached 9 points. Recovery time; record the time from onset to recovery as sedation time.
(3) Sedation degree: 10 minutes after the child wakes up after the operation, the Ramsay sedation score^7^ scale was used to evaluate the postoperative sedation degree of the child, which was divided into 6 grades from 1 to 6 points:, Irritability; 2: quiet cooperation; 3: drowsiness, can follow instructions; 4: sleep, but can be awakened; 5: sluggish breathing; 6: deep sleep, unable to wake up. Among them, 2-4 points for good sedation, and 5-6 points for excessive sedation.
(4) The disparitys in cognitive function between the two sets of children before and after sedation were contrast, and the Montreal cognitive assessment (MoCA) scale^8^ was used for testing. MoCA consists of 8 cognitive domains and 11 examinations, covering visuospatial and executive function, naming, attention, computation, language, abstraction, delayed recall and orientation. With a total score of 30 points, the scale is administered by trained physicians in a quiet ward and is limited to 10 minutes to complete. There are a total of 30 questions, each correct answer will be scored 1 point, wrong answer or unknown answer will be scored 0 point, the total score is 0∼30 points. 27-30 points: normal; 21-26 points: mild impairment; 10-20 points: moderate impairment; 0-9 points: severe impairment.
(5) Record whether there are adverse reactions, such as bradycardia, respiratory depression, nausea, vomiting, hypoxia, irritability, etc.
(6) To analyze the correlation between the degree of sedation and postoperative cognitive function in children.

### Statistical analysis

In this examination, all the data are sorted, and a corresponding database is established for it, and all the databases are entered into Statistical Product and Service Solutions (SPSS) 26.0 for data processing (IBM, Armonk, NY, USA), and the measurement data is tested for normality, and the expression method is as follows: (x̅ ± s), In line with normality, the multiple-set test is independent-sample t-test for between-set data, and paired-sample t-test for intra-set data; the rate is expressed as %, and the test is χ2; correlation is analyzed by Pearson; when P<0.05, the data is considered to be between The disparity is statistically extensive.

## Results

### Contrast of heart rate, blood pressure, and pulse oxygen saturation between the two sets of children

After the two sets of children completed nasal sedation, there was no extensive disparity in the heart rate, blood pressure and pulse oxygen saturation of the children in the T0-T3 time period when entering the room (P>0.05), as shown in Table 1.

**Table 1:** Contrast of heart rate, blood pressure and pulse oxygen saturation in two sets of children

Time	Indicator	Contrast set(n=45)	Examination set (n=45)
TO	Heart rate(beats/min)	89.82±3.16	90.49±4.63
	Pulse oxygen saturation(%)	96.85±3.44	97.73±3.59
	Mean arterial pressure(mmHg)	60.56±4.93	62.52±5.15
T1	Heart rate(beats/min)	89.96±3.36	91.56±4.57
	Pulse oxygen saturation(%)	97.55±3.52	98.15±3.26
	Mean arterial pressure(mmHg)	64.23±4.26	66.51±5.37
T2	Heart rate(beats/min)	90.15±3.71	92.43±4.52
	Pulse oxygen saturation(%)	97.45±3.59	98.49±3.92
	Mean arterial pressure(mmHg)	65.59±4.82	66.71±5.38
T3	Heart rate(beats/min)	93.52±3.16	94.51±4.94
	Pulse oxygen saturation(%)	99.86±3.44	99.73±3.619
	Mean arterial pressure(mmHg)	66.81±5.79	67.82±5.37

### Contrast of the onset time, recovery time and sedation time of the two sets of children

The onset time of sedation in the study group was significantly lower than that in the control group, and the sedation time was notoriously higher than that in the contrast set (P<0.05), See Table 2.

**Table 2:** Contrast of onset time, wake-up time and sedation time of two sets of children

set	Onset time (s)	Sedation time (min)	Recovery time (min)
Contrast set(n=45)	80.23±10.66	14.15±5.26	61.53±10.49
Examination set (n=45)	29.53±8.32	21.53±5.19	27.57±9.16
t	10.596	7.563	8.639
P	<0.001	<0.001	<0.001

### Contrast of Ramsay score of sedation in two sets of children

The Ramsay score of sedation in the two sets was contrast, and it was found that the Ramsay score of the examination set was notoriously higher than that of the contrast set, and the disparity was statistically extensive (P<0.05), as shown in Table 3.

**Table 3:** Contrast of Ramsay score of sedation in two sets of children

Set	Ramsay Score (Minute)
Contrast set (n=45)	1.43±0.53
Examination set (n=45)	2.47±0.69
t	11.863
P	<0.001

### Contrast of cognitive function between the two sets before and after sedation

Before sedation, the MoCA scores of the two sets of children were higher, and the study group was significantly lower than the control group (P>0.05). See Table 4.

**Table 4:** Contrast of cognitive function of two sets of children before and after sedation

Set	Before sedation	After sedation
Contrast set(n=45)	28.53±1.53	26.53±2.33
Examination set(n=45)	28.15±1.87	24.25±2.12
t	0.596	6.235
P	0.513	<0.001

### Contrast of the incidence of adverse reactions after sedation between the two sets

After sedation, there was no significant difference in the incidence of adverse reactions between the two groups (P>0.05), as shown in Table 5.

**Table 5:** Contrast of the incidence of adverse reactions after sedation in the two sets [n (%)]

Set	Bradycardia	Respiratory depression	nausea	Vomit	Hypoxia	Irritable	Total incidence
Contrast set (n=45)	1	1	1	1	1	2	7(15.56)
Examination set (n=45)	3	0	0	1	1	1	6(13.33)
χ^2^							0.963
P							0.519

### Correlation analysis between the degree of sedation and postoperative cognitive function

**Figure 1:**
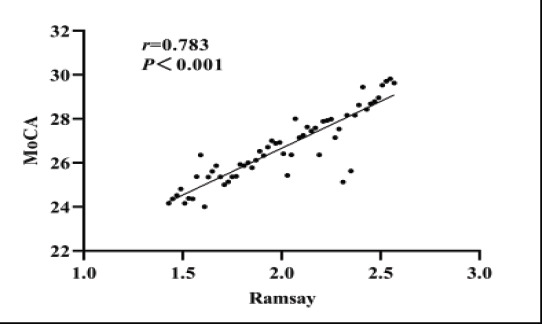
Correlation between Ramsay score and MoCA score

After the completion of sedation, the Ramsay score and MoCA score of the two sets were collected. The correlation results showed that the Ramsay score and MoCA score were notoriously positively correlated (P<0.05), as shown in Figure 1.

## Discussion

In pediatric hospitals, inguinal hernias are one of the most common infections, with an incidence of up to 4%. The high ligation of the hernia sac requires the removal of the external oblique aponeurosis, which is then removed after the high ligation of the hernia sac. This surgical method is difficult to operate, which will lead to the destruction of the anatomical structure of the inguinal canal, and may also cause the muscles of the inguinal canal, cremasteric Muscles, blood vessels and nerves and other peripheral tissues have sudden diseases, and it is also very likely to accidentally injure peripheral tissues, such as cremaster muscle, spermatic blood vessels and nerves, and the incidence of postoperative complications in children is high9. In addition, most of the children who are examined will be afraid and worried because of the abnormal state of the hospital, which makes the children unable to cooperate with the examination. Therefore, for better coordination and better completion of the test, infants and young children should receive sedation to reduce their fear and anxiety and enhance surgical cooperation^10^.

At present, the development and progress of minimally invasive surgery have greatly reduced a series of problems caused by traditional surgical methods. There are many clinical application cases and advantages of this surgical method. In the clinical therapy of inguinal hernia in children, laparoscopic Hernia sac high ligation is the preferred surgical plan at this stage, and it has gradually replaced the traditional laparotomy. Moreover, the operation of incising the sufferer's external oblique aponeurosis in traditional surgery is avoided, so the anatomical structure of the inguinal canal will not be damaged, thereby avoiding damage to the spermatic nerve and blood vessels^2^. In contrast, traditional laparotomy has many disadvantages, such as extensive intraoperative incision and long operation time.

In this study, the mean arterial pressure and heart rate of the two groups of children were observed immediately after entering the operating room (TO), 5 minutes (T1), 10 minutes (T2), 15 minutes (T3) and 20 minutes (T4) after entering the operating room blood oxygen saturation, after entering the recovery room, blood oxygen saturation, the results showed that after the two sets of children completed nasal sedation, there was no extensive disparity in the heart rate, blood pressure and pulse oxygen saturation of the children in the T0∼T3 time period when entering the room (P>0.05). The main reason is that dexmedetomidine is a highly selectivea2 adrenergic receptor agonist, which achieves a sedative and hypnotic effect similar to natural non-rapid eye movement sleep in humans by binding to the centrala2 adrenergic receptor. And it has analgesic, sedative, anti-sympathetic and other effects, and no respiratory depression, has been widely used in pediatric sedation^11^. Dexmedetomidine intranasal administration is currently a commonly used outsufferer sedation method, however, the drug may cause bradycardia or hypotension in children. It is also more common in outsufferer sedation^12^. Ketamine is a classic anesthetic with sedative and analgesic properties, however, it has some adverse side effects including nausea, hypertension, and tachycardia^13^.

Administration can be done in a variety of ways, including nasal administration. This examination also found that the onset time of sedation in the examination set was notoriously higher than that in the contrast set, the sedation time was notoriously higher than that in the contrast set, the Ramsay score was notoriously higher than that in the contrast set, The MoCA score of the study group was significantly lower than that of the control group (P<0.05); The reason for the analysis is mainly because esketamine is the dextro-enantiomer of ketamine, and the potency is about twice that of ketamine. At the same time, contrast with ketamine, esketamine has faster recovery, less postoperative pain, faster cognitive function recovery, and lower incidence of mental side effects, and esketamine can offset the bradycardia and hypotensive effects of dexmedetomidine^14^,^15^. Therefore, the combination of esketamine and dexmedetomidine may be beneficial in children. At present, the application of esketamine combined with dexmedetomidine in children's sedation, especially the examination of esketamine combined with dexmedetomidine nasal drops has not been seen^16^,^17^. However, this examination confirmed that dexmedetomidine combined with esketamine nasal drops for sedation in children has the advantages of stable hemodynamics. Contrast with single use of midazolam anesthesia, the onset time is shorter and the sedative effect is better.it is good. After sedation, there was no significant difference in the incidence of adverse reactions between the two groups (P>0.05); Adrenoceptor agonists, the dextroisomer of the racemic mixture medetomidine, have good sedation in awake sufferers and can produce a sedative state similar to phase II sleep^18^. Bian's^19^ examination found that it can also reduce the incidence of postoperative delirium. When used in conjunction with other sedative and analgesic drugs, it can better exert the synergistic effect of analgesia and sedation, and can notoriously reduce the amount of analgesic drugs, thereby reducing the need for surgery. Adverse reactions of emotional instability in sufferers after^20^. Although this examination has achieved certain examination results, there are still some limitations, because the current application of esketamine combined with dexmedetomidine in children's sedation, especially the examination on esketamine combined with dexmedetomidine nasal drops. It has not been seen yet. As a relatively novel examination, this examination has selected relatively few examination objects and has no other literature for reference. It may have certain deficiencies in methods and other aspects. Therefore, in future examination, it is necessary to further expand the sample size and The selection range of the examination subjects was further analyzed, and the efficacy of dexmedetomidine combined with esketamine combined with intranasal infusion before laparoscopic high ligation of hernia sac in infants and young children was further analyzed.

To sum up, for infants and young children undergoing laparoscopic high ligation of hernia sac, dexmedetomidine combined with esketamine nasal drops can notoriously improve the sedation, reduce the incidence of adverse reactions, and It will not damage the cognitive function of children, and has high clinical effect and safety, which is worthy of clinical promotion.
